# Process for a Reactive Monomer Alignment Layer for Liquid Crystals Formed on an Azodye Sublayer

**DOI:** 10.3390/ma11071195

**Published:** 2018-07-12

**Authors:** Junren Wang, Colin McGinty, Robert Reich, Valerie Finnemeyer, Harry Clark, Shaun Berry, Philip Bos

**Affiliations:** 1 Liquid Crystal Institute, Kent State University, Kent, OH 44242, USA; jwang45@kent.edu (J.W.); cmcginty@kent.edu (C.M.); 2 Lincoln Laboratory, Massachusetts Institute of Technology, Lexington, MA 02421, USA; reich@ll.mit.edu (R.R.); Valerie.finnemeyer@ll.mit.edu (V.F.); hrclark@ll.mit.edu (H.C.); sberry@ll.mit.edu (S.B.)

**Keywords:** photoalignment, liquid crystals, reactive monomers, azo dye

## Abstract

In this work, the detailed studies of surface polymerization stabilizing liquid crystal formed on an azodye sublayer are presented. The surface localized stabilization is obtained by free-radical polymerization of a dilute solution of a bi-functional reactive monomer (RM) in a liquid crystal (LC) solvent. To optimize the process for surface localized stabilization, we investigate the effects of several process parameters including RM concentration in LC hosts, the types of materials (either RM or LC), the photo-initiator (PI) concentration, ultra-violet (UV) polymerization intensity, and the UV curing temperature. The quality of surface localized stabilization is characterized and/or evaluated by optical microscopy, electro-optical behavior (transmission/voltage curve), the life test, and photo-bleaching. Our results show that, by carefully selecting materials, formulating mixtures, and controlling the polymerizing variables, the RM polymerization can be realized either at the surface or through the bulk. Overall, the combination of surface localized stabilization and photo-alignment offers an elegant and dynamic solution for controlling the alignment for LC, which could play a profound role in almost all liquid crystal optical devices.

## 1. Introduction

Well-aligned liquid crystal (LC) layers are required in almost all their electro-optic applications. Currently, the dominant liquid crystal alignment method–mechanically rubbed polyimide suffers several problems such as the creation of contaminating particles, scratches, and electrostatic charges [1]. Therefore, alternative non-contact techniques to align liquid crystals are preferred. As one of the most promising non-contact alignment methods, photo-alignment can utilize the polarized light to generate the anisotropy on the substrate surface, which overcomes the problems mentioned above [2,3,4,5,6]. Photo-alignment based on azo-dye offers an intriguing way to fabricate liquid crystal devices due to the low cost as well as the ability to create complex and precise patterns under mild conditions [5,7]. One drawback of the azo dye photo-alignment layer is its instability to subsequent exposure to light. To stabilize the initial alignment for liquid crystals, solutions including the polymerizable azodyes [8] and passive reactive monomer layer spin-coated on the top of the azo-dye sublayer [9] have been explored. To simplify the process, V. Finnemeyer et al. [6] proposed that a small amount of reactive monomer added to the liquid crystal host could provide excellent stabilization of the alignment by phase separation. Additionally, C. McGinty et al. [10,11] showed that the underlying azodye layer can be photo-bleached to eliminate its visible absorption as well as its ability to re-orient further. 

Depending on the location where the full polymerization of the reactive monomer (RM) completes from a low molecular weight liquid crystal solvent, the formed structure can be generally classified as a bulk polymer network or a surface localized polymer. Most prior studies were focused on the polymer network that stabilized LC on a rubbed polyimide layer and have shown that some parameters such as solubility parameters of RM in LC [12], the RM concentration in LC [13,14], LC materials [15,16], reactive monomers [17], ultraviolet (UV) curing intensity [18], and the UV curing temperature [19,20] could affect the resulting morphology and subsequent electro-optical behaviors. I. Dierking et al. reported that monomer solubility played a primary role in determining network morphology in the polymer stabilized liquid crystal (PSLC) [12]. Poorly soluble monomers form coarse “rice grain like” structures while soluble monomers yield smooth and continuous networks. R. Yamaguchi et al. reported that the morphology of polymer stabilized liquid crystal cells can be changed by selecting liquid crystal materials. Using an LC with a tolane substance, a “rice grain like” morphology can be obtained and can lower the driving voltage [15]. S. Hudson and L.C. Chien studied the morphology of PSLC when polymerized at different conditions (such as UV intensity and different temperatures) [19,21] and, in the case of different reactive monomers, at isothermal conditions [22]. More work related to PSLC can be found from several review papers by A. Sonin and N. Churochkina [23] and I. Dierking [24,25,26].

The existence of the polymer network in PSLC can introduce light scattering due to the refractive index mismatch between the bulk polymer network and liquid crystal [18], which will undermine the quality of the display. To eliminate light scattering, surface localized polymerization is preferred. Previous approaches for polymerizing the reactive monomer on the surface instead of in LC bulk include (1) selecting RM materials with high UV absorption [27]; (2) using a phase-separated composite film (PSCOF) [28,29], which is formed due to slow polymerization, phase separation, and fast diffusion of small molecules; and (3) utilizing the electric field to localize RM to the surface [30]. However, many aspects of the process to achieve and to optimize the surface localization of the polymer layer on a photo-alignment sublayer have not been explored.

To have a better understanding as well as a better control over the surface localized stabilization on a photo-alignment sublayer, it is necessary to study the process variables such as materials and UV polymerization conditions. In this paper, we begin these investigations in detail. Optical microscopy observations, transmission-voltage curves, life-test, and photo-bleaching are utilized to help characterize and evaluate the quality of the surface stabilized liquid crystals. 

## 2. Experimental

### 2.1. Sample Preparation

**Figure 1:**
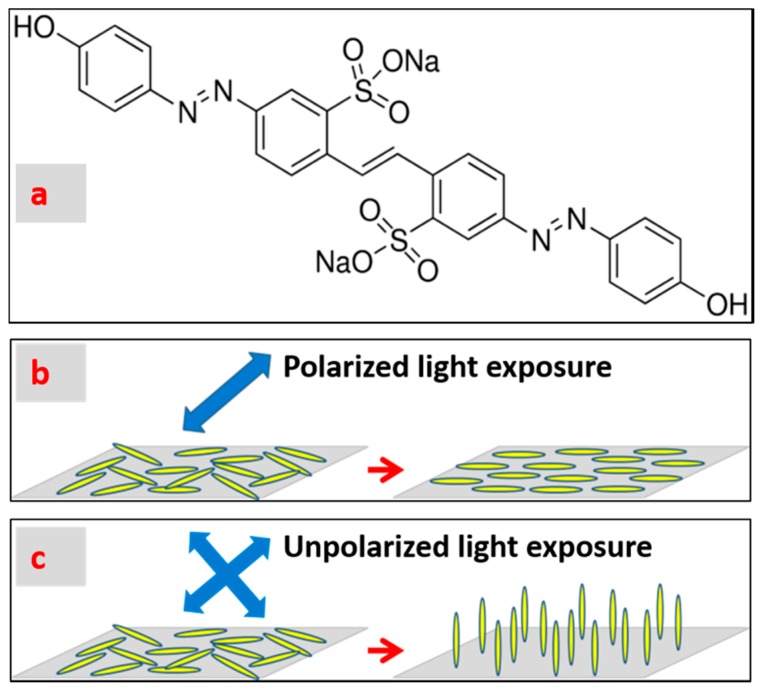
(**a**) Molecular structure of brilliant yellow. (**b**) When exposed to polarized light, brilliant yellow molecules undergo in-plane re-orientation to a direction of 90 degrees with respect to the light polarization direction. (**c**) When exposed to un-polarized light, brilliant yellow molecules undergo out-of-plane re-orientation to a direction along to the light propagation direction.

At 22 ± 1 °C and 20 ± 3% relative humidity (RH), 1 wt.% brilliant yellow (BY, CAS #: 3051-11-4, obtained from Sigma Aldrich, St. Louis, MO, United States) dissolved in dimethylformamide (DMF, 99.8% anhydrous, from Sigma Aldrich) was spin-coated onto UV/Ozone cleaned indium tin oxide (ITO, Corning Inc., NY, USA) glass substrates (1 inch by 1 inch) at 1500 rpm for 30 s to obtain a uniform and a thin BY layer [31]. After baking at 90 °C for 10 min to evaporate solvent, the BY-coated glass substrates were assembled into cells using spacers of 5 µm thickness. The cell was then sealed on two sides with UV curable optical adhesives (NOA65, Norland Products Inc., Cranbury, NJ, USA) to create a channel for capillary-filling of LC mixtures. Then the assembled cells were exposed to linearly polarized blue light (Luxeon Royal blue LED with peak wavelength of 447 nm) at 25 mW/cm^2^ for 10 min to obtain a uniform planar alignment. The molecular structure of brilliant yellow is shown in Figure 1a and its absorbance spectra and photo alignment mechanism are explained in a previous research study [31,32,33]. Its photo-orientations to polarized light [31] and un-polarized light [10] are sketched in Figure 1b,c, respectively.

Following the photo-alignment, the cells were capillary-filled with mixtures of RM and photo-initiator (PI) dissolved in the LC host at a temperature of 10 °C above LC’s clearing points. Then the filled cells were exposed to 365 nm UV light to polymerize the reactive monomer.

Two reactive monomers, 2-Methyl-1,4-phenylene bis(4-(3-(acryloyloxy)propoxy)benzoate) (RM257) from Merck and Bisphenol A dimethacrylate (Bis-MA) from Sigma Aldrich, and two kinds of liquid crystals known as the cyano eutectic liquid crystal (E7, clearing point = 60 °C, birefringence Δn = 0.217 at 589 nm and 20 °C) and the super fluorinated liquid crystal (ZLI-4792, clearing point = 92 °C, birefringence Δn = 0.0969 at 589 nm and 20 °C) from Merck were chosen to build different RM/LC combinations. In addition, a small amount of 2,2-Dimethoxy-2-phenylacetophenone (Irgacure 651) from Sigma Aldrich was added as a photo-initiator.

Effects of six process variables for surface localized stabilization including RM concentration, types of LC, types of RM, PI concentration, UV curing intensity, and the UV curing temperature were investigated by changing one parameter at a time. Accordingly, different samples/cells were prepared as shown in Table 1, Table 2, Table 3, Table 4, Table 5 and Table 6.

### 2.2. Alignment and Stabilization Examination

The cells filled with mixtures of RM and LC were checked for their alignment by placing them between cross polarizers. The homogeneous (or planar) alignment is assured when the cell appears between alternative dark and bright light under crossed polarizers when the cell is rotated with respect to the polarizer(s) while the homeotropic alignment is secured when the cell appears dark under crossed polarizers regardless of the rotation.

The stabilization of LC was checked with the life test, photo-bleaching, and the electro-optical performance (transmission versus applied voltage curves, also called T/V curve). T/V curves were collected with a green light source (550 nm) and compared both before and after UV polymerization of the reactive monomer. The transmission is measured using a photodetector (Thorlabs Inc., Newton, NJ, USA) and the transmission is represented by the voltage shown on the photodetector. Life tests re-expose the LC-filled cells to linearly polarized blue light at 45° with respect to the original photo-alignment process. Photo-bleaching re-exposes the cells with un-polarized blue light to eliminate the photosensitivity of the azo dye layer [10,11] because the brilliant yellow molecules re-orient along the un-polarized light propagation direction where no light absorption occurs and the molecules are at rest. If the monomer was not polymerized on (or close to) the surface, the resulting cells would show: (1) re-alignment with life test and/or photo-bleaching, (2) some light scattering observation because of the network through bulk, or (3) increased threshold voltage as indicated by the T/V curve after UV polymerization.

## 3. Results

The effects of parameters (including RM concentration, types of LC, types of RM, photo-initiator concentration, UV curing intensity, and UV curing temperature) on surface localized stabilization were characterized and evaluated based on macroscopic observation, life test, photo-bleaching, and measured T/V curves. These effects are presented below.

### 3.1. Effects of RM Concentration

**Figure 2:**
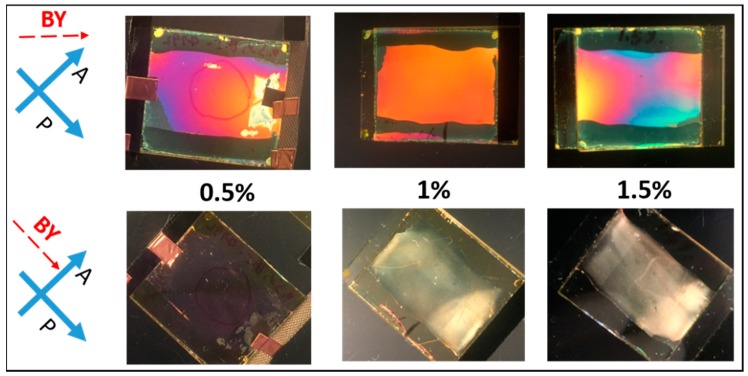
Observation of cells between crossed polarizers after UV polymerization. Cells were filled with mixtures of different RM (Bis-MA) concentration (0.5%, 1%, or 1.5%) in LC (ZLI-4792).

After photo-alignment with linearly polarized blue light, cells filled with mixtures of different concentrations of RM (Bis-MA) dissolved in LC (ZLI-4792) show planar alignment along the BY alignment both before and after polymerization, which is shown in Figure 2. The observation of different colors indicates that the gap thickness of these home-made cells is not very uniform. These cells appeared bright when the BY alignment direction was 45° to either polarizer direction and appeared dark when the BY alignment direction was parallel or perpendicular to one polarizer direction. It is significant that light scattering was observed for cells with a higher RM concentration (both 1% and 1.5%) while the lower RM concentration (0.5%) did not show the light scattering, which is shown in Figure 2.

**Figure 3:**
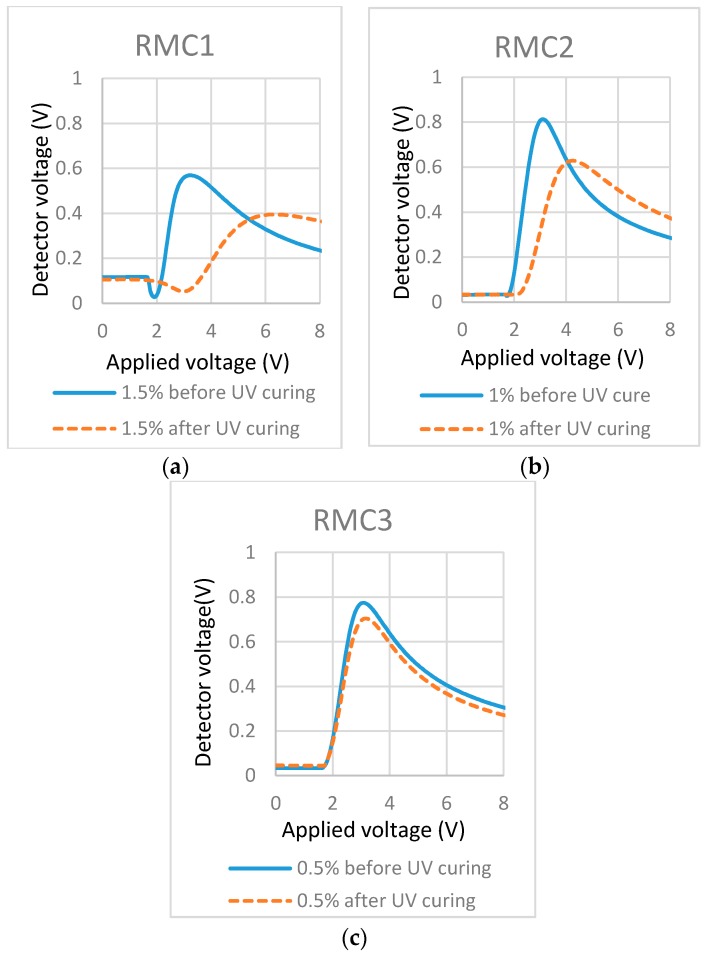
Transmission-applied voltage curves of cells filled with mixtures of different concentrations of RM in LC. (**a**) 1.5% Bis-MA in LC; (**b**) 1% Bis-MA in LC; and (**c**) 0.5% Bis-MA in LC.

Previous studies showed that the formation of the bulk polymer network would lead to light scattering [18] and the increase of the threshold voltage [35]. Therefore, we believe the observed light scattering is ascribed to the formation of the bulk polymer network. This is also confirmed in the measured T/V curves (Figure 3a,b) where the curve peaks and threshold voltages (dash line curve) showed the right-shift after UV polymerization compared with the right-shift before UV polymerization (solid line curve). For the lower RM concentration (0.5%), no increase in threshold voltage (Figure 3c) and no observed light scattering (Figure 2) indicated the formation of the polymer layer at (or near to) the substrate. To further evaluate the localized surface and its stabilization of RM, we conducted the life test and photo-bleaching experiment on the cell filled with 0.5% RM in LC.

**Figure 4:**
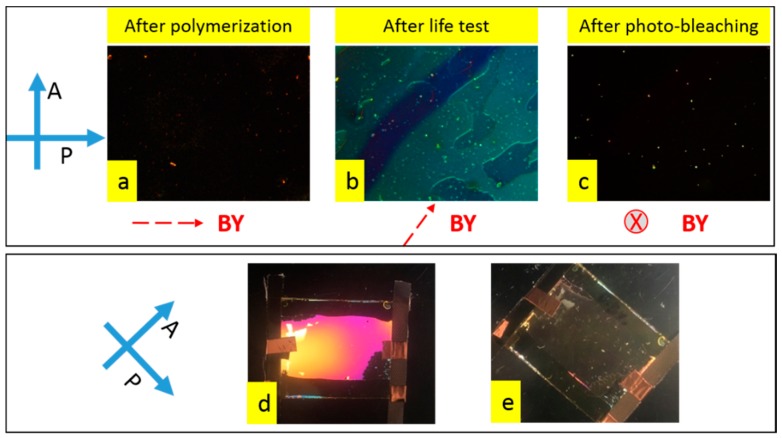
Polarized microscope images of the cell (0.5% cell in Figure 2): (**a**) before life-test; (**b**) after life test; and (**c**) after photo-bleaching. The BY alignment was represented by a red dash arrow with a dynamic re-orientation direction after the life-test and photo-bleaching with respect to the polarizer and/or analyzer direction. Macroscopic observation of cells after photo-bleaching when the original LC alignment was 45° to polarizer (**d**) or parallel to one polarizer (**e**).

As shown in the microscopic images of Figure 4, in the afterlife test, the BY realigned and the cells showed some brightness (blue/green appearance) between crossed polarizers (Figure 4b). The birefringence magnitude of this BY (tens of nanometer thickness [34]) is considerably less than the aligned nematic LC (close to 5 µm). Followed by photo-bleaching (exposed to un-polarized light), the BY was realigned perpendicularly to the substrates and the cell appeared dark between crossed polarizers (shown in Figure 4c), which is similar to the original appearance before the life-test (Figure 4a). Although the BY layer showed some realignment, the alignment of liquid crystal in the cell was not changed, which is shown in Figure 4d,e. This is the same as the cell before the life-test and photo-bleaching (0.5% cell shown in Figure 2). Combining Figure 4 and the T/V curve in Figure 3, we can conclude that: (1) the polymerization occurred at (or close to) the substrate surface in the cell containing lower concentration (0.5%) of RM in LC while the higher concentration (1%, 1.5%) led to the formation of the bulk polymer network; (2) the formed surface polymer layer in 0.5% concentration cell worked like an alignment layer between BY and LC, which preserved the LC alignment regardless of the reorientation of BY.

### 3.2. Effects of Types of LC

Depending on the applications, liquid crystals with specific properties (such as proper birefringence, dielectric constant, clearing points, ion solubility, etc.) would be preferred. In this section, two different liquid crystals were chosen for the study. One is a common cyano eutectic liquid crystal mixture–E7 and the other one is super fluorinated liquid crystal–ZLI-4792. Two cells filled with mixtures contain 1.5% RM257 dissolved in either E7 or ZLI-4792. These two cells were processed through polymerization at the same conditions.

**Figure 5:**
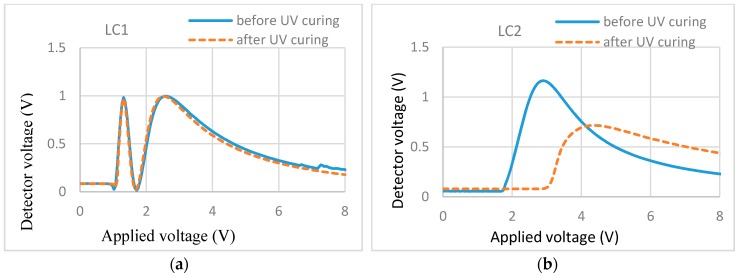
T/V curves of (**a**) 1.5% RM257 in E7 and (**b**) 1.5% RM257 in ZLI-4792.

The cell filled with RM257 (1.5%)/E7 showed no light scattering and no increase in the threshold voltage, which is illustrated in the T/V curves of Figure 5a. The cell filled with RM257 (1.5%)/ZLI-4792 showed light scattering and some increase in the threshold voltage (Figure 5b). It may be concluded that 1.5% RM257 formed surface localized stabilization in the E7 cell while it formed a bulk network in the ZLI-4792 cell. The difference indicates that the types of LCs counts when using the reactive monomer to stabilize the alignment. With regard to the analysis, we will propose a possible explanation in the Discussion section.

### 3.3. Effects of RM Types

**Figure 6:**
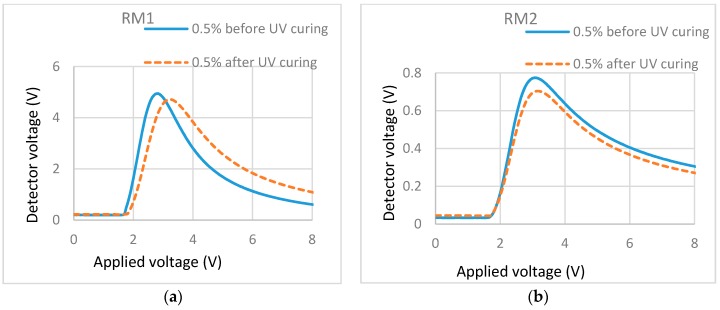
T/V curves of cells containing different monomers in the same LC. (**a**) 0.5% RM257 in ZLI-4792; (**b**) 0.5% Bis-MA in ZLI-4792.

As shown above, the types of LCs showed a difference. To see whether the types of reactive monomer matter or not, we conducted experiments with a different RM in the same LC. The results were compared and explained using T/V curves, which is shown in Figure 6.

In Figure 6a, the T/V curve (dash line) after polymerization shifted to the right when compared with the T/V curve before polymerization (solid line). This showed that, for the cell filled with 0.5% RM257 in ZLI-4792, there was a small increase in the threshold voltage and weak light scattering after polymerization. These indicate that there were some formations of continuous networks through bulk. T/V curves (Figure 6b) for cells filled with 0.5% Bis-MA in ZLI-4792 did not show an increase in the threshold voltage and showed almost no light scattering, which was macroscopically observed. This is shown in the 0.5% cell in Figure 2. With these results, we can conclude that, similar to the types of LC, the types of RM also have effects on the localized surface stabilization.

### 3.4. Effects of Photo-Initiator (PI) Concentration

The major role of the photo-initiator in radical polymerization is to create reactive species (free radicals) under mild conditions (such as UV light exposure, room temperature) and promote radical reactions. In order to investigate its effects, cells filled with mixtures of 0.3% RM257 in ZLI-4792 with different amounts of added PI were studied.

**Figure 7:**
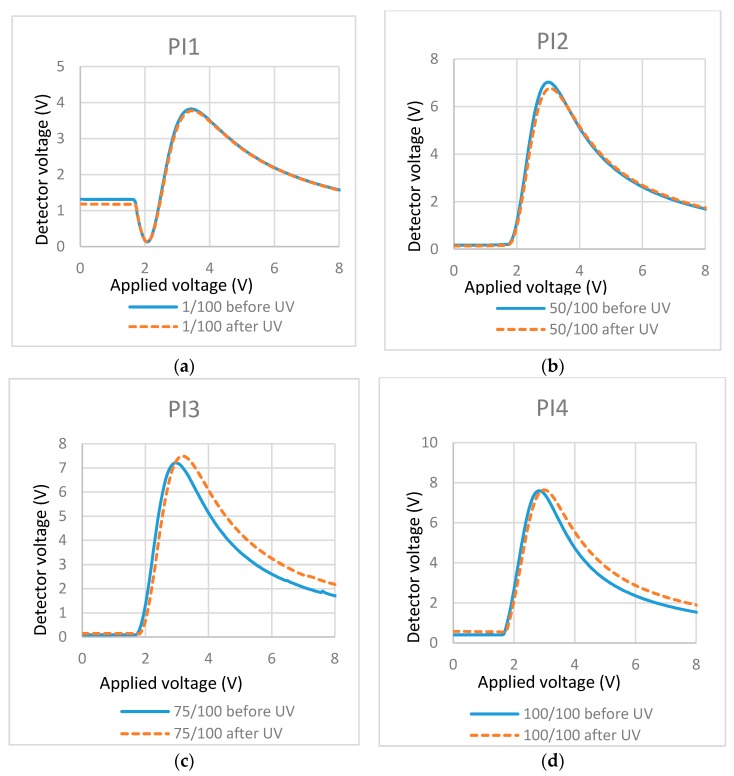
T/V curves of cells containing mixtures with different concentrations of photo-initiator (PI): (**a**) PI/RM = 1/100; (**b**) PI/RM = 50/100; (**c**) PI/RM = 75/100; and (**d**) PI/RM = 100/100.

As shown in Figure 7a,b, at a lower PI concentration (PI/RM = 1/100 and 50/100, respectively), T/V curves almost kept the same before and after UV polymerization. As the PI concentration increased, the threshold voltage increased slightly and weak light scattering was observed, which is shown in the T/V curves in Figure 7c,d. These results may indicate that, at a lower PI concentration, the RM tends to polymerize on the surface and the RM may possibly polymerize with some protrusion to the bulk at a higher PI concentration.

### 3.5. Effects of UV Curing Intensity

**Figure 8:**
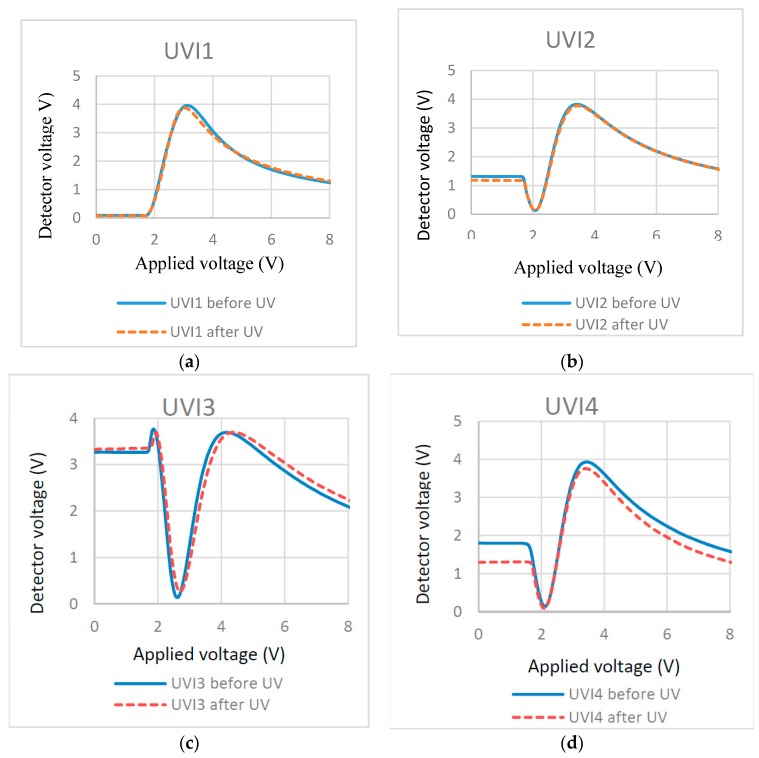
T/V curves of cells cured at different UV exposure intensity and time: (**a**) 0.75 mW/cm^2^ for 70 min; (**b**) 3.5 mW/cm^2^ for 10 min; (**c**) 21 mW/cm^2^ for 2.5 min; and (**d**) 21 mW/cm^2^ for 10 min.

To investigate the effects of UV light intensity on the surface localized stabilization, we polymerized cells filled with 0.3% RM257 in ZLI-4792 with different UV intensities. Their effects were illustrated in T/V curves (Figure 8) before and after polymerization.

Note that the plots look somewhat different when compared before and after UV curing. This may be due to the non-uniform thickness of the home-made cells. A different area may be characterized before and after UV curing, which leads to the difference of the measured retardation (detector voltage). Despite the small difference of the T-V curve, the threshold voltage from the T-V curves (measured before and after curing) offer more important information to tell the location of the RM formed polymer layer. If the threshold voltages remain at similar levels before and after curing, this can determine the localized surface RM polymerization. If the threshold voltages are clearly different, it means the polymer network forms through the LC bulk [35]. The plots in Figure 8 show that no effect is found on the threshold voltage after polymerization, which indicates that UV intensity does not greatly affect the surface localized stabilization. This may be because the UV intensity does not affect the production of radical species of photo-initiator or does not affect the chain reaction rate. To further understand the effects in detail, the high-resolution scanning electron microscopy (SEM) may be used to check the formed polymer morphology.

### 3.6. Effects of UV Curing Temperature

The UV curing temperature has an effect on the long-range order of the LC solvent. To see whether the stabilization is affected by the disorder in the LC system, we conduct experiments of polymerizing cells (filled of 1.5% RM257 in E7) at different temperatures.

**Figure 9:**
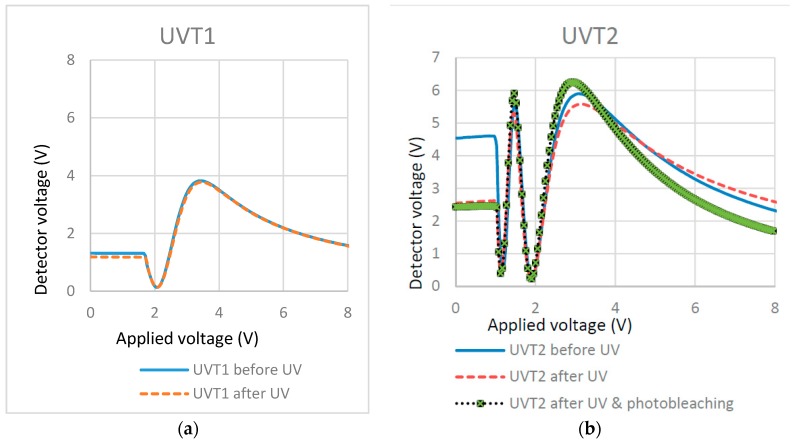
T/V curves of cells cured at different temperatures: (**a**) 22 °C and (**b**) 80 °C.

**Figure 10:**
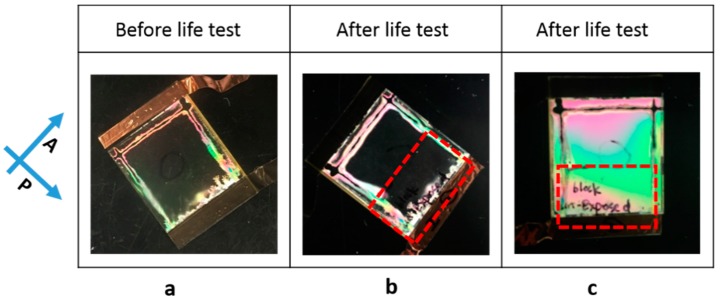
Macroscopic observation of the cell before photo-bleaching (**a**) and after photo-bleaching (**b**,**c**). The original LC alignment was parallel to one polarizer (**b**) or 45° to one polarizer (**c**). Half of the cell (highlighted in the dashed line box of **b**, **c**) was masked during the exposure. Therefore, a uniform dark state across the cell indicates stable alignment.

As shown in Figure 9a, the T/V curves before and after polymerization are similar for the cell cured at a low temperature (22 °C). For the cell cured at a high temperature (80 °C), the T/V curves show a difference in speed before and after polymerization. However, the threshold voltage is almost the same, which is shown in Figure 9b. Another T-V curve measurement after photo-bleaching (exposed to un-polarized light for 8 h) appears similar to the one before photo-bleaching and doesn’t show an increased threshold voltage. Macroscopic observation between crossed polarizers revealed that the cell cured at 80 °C kept its original alignment and there was no light scattering observed, which is shown in Figure 10. All these indicate that RM localized near the surface and stabilized the liquid crystal alignment regardless of the re-orientation of the azodye sublayer. Furthermore, it would be interesting to point out the stabilization of the cell cured at 80 °C in terms of where the temperature is actually above the clearing point of E7 (which is 60 °C). Even though the UV polymerization occurred at an isotropic state, the original LC alignment was preserved. This indicates that RM monomers/oligomers were diffused to the surface and were aligned by the azodye sublayer while undergoing the polymerization process.

## 4. Discussion

As seen from the results above, the concentrations of reactive monomers and the types of liquid crystals show larger effects on the surface localized polymerization compared to other parameters. These show some hints on the physical properties of RMs and LCs such as the solubility limit [12].

The solubility limit can be compared through the solubility parameters. For a material with a known molecular structure, its solubility parameter can be calculated based on the group contribution theory. In our case, most materials (either RM or LC) have known molecular structures except for ZLI-4792, which is not released by Merck. However, based on the molecular structures of some super fluorinated liquid crystals similar to ZLI-4792, we performed some estimated calculations for RM and LC.

**Figure 11:**
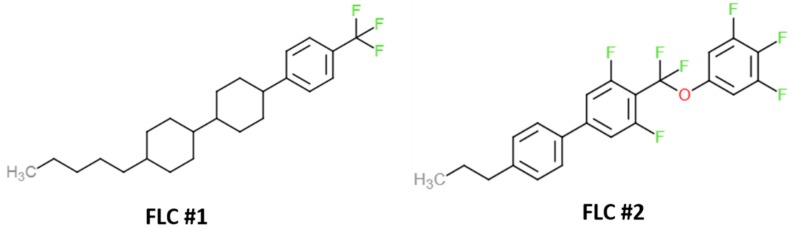
Molecular structures of two fluorinated liquid crystals (FLC) chosen to estimate the solubility parameters for ZLI-4792.

According to the components in the molecular structure, the cohesive energy, and molar volume obtained from the Polymer Handbook [36], solubility parameters (δ) of two fluorinated liquid crystals (FLC, as shown in Figure 11) were calculated using group contribution theory, which is shown in Table 7 and Table 8, respectively.

The estimated solubility parameters of RMs and LCs used in experiments are presented in Table 9. We can see that the fluorinated liquid crystals have the smallest solubility parameter values. The smaller difference in the solubility parameters between two materials refers to higher solubility while the larger difference refers to lower solubility.

**Figure 12:**
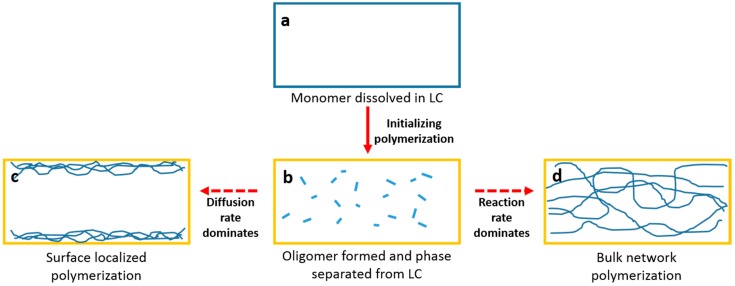
Sketched mechanism for the polymerization of RM from LC solvent: (**a**) RM dissolved in LC before UV exposure; (**b**) Oligomer formed and phase separated from LC after initial UV polymerization; (**c**) Surface localized polymerization and (**d**) bulk network polymerization.

The sketch in Figure 12 may explain the possible mechanism of the surface localized polymerization of RM from the LC solvent. Before UV polymerization or exposure, the RM and PI are homogeneously dissolved in LC (Figure 12a). At the initial UV exposure (polymerization), photo-initiator molecules absorb UV photons and produce radical species. The free radicals will promote several bi-functional RM molecules to form oligomers by using a chain reaction. The oligomers are not very soluble to some extent and would decline when separated from the LC solvent (Figure 12b). As the oligomer increase its molecular weight through further chain reactions, the insolubility would repel the LC solvent by diffusing to the substrates while the chain reaction continues. If the diffusion rate dominated, the oligomer would be localized at (or close to) surface (Figure 12c), which is followed by a further polymerization reaction. If the polymer chain reaction dominated, the oligomer that would not have enough time to move the surface would undergo another chain reaction at its initial location, which would form the bulk polymer network (Figure 12d). In both cases, the final polymer would have preferred alignment because of the bi-functionality of the reactive monomer and the long-range order in the LC solvent aligned by the azodye sublayer. Previous studies on the polymer stabilized liquid crystal have described the effect of the diffusion rate versus the reaction rate in the formation of the polymer strand morphology [19,21,27]. A more bulk network will be formed if the reaction rate dominates over the diffusion rate. In our case, a similar result to the one proposed in Figure 12 should be discovered.

Considering the effects of RM concentration and the effects of different LCs (such as E7 versus ZLI-4792) and different RMs (such as RM257 versus Bis-MA), the underlying reason for the differing final morphology appears to be the solubility limit. If the monomer has a smaller solubility, the corresponding oligomer would have a smaller solubility limit. In this case, the oligomer may be phase separated in the bulk to have local areas of higher density that would promote polymerization in the bulk. Essentially, the higher solubility limits may not have a high enough local density in the bulk to react. Therefore, the oligomer can diffuse to the surface where it achieves a higher density and is able to realize surface localized polymerization.

## 5. Conclusions

We have shown that process parameters (such as RM concentration, types of LC, types of RM, photo-initiator concentration, UV curing intensity, and UV curing temperature) could somewhat influence the surface localized stabilization. By tuning these parameter(s), RM polymerized morphology can vary from the surface localization to the bulk network. Using optical microscopy, T/V curves, the life test, and photo-bleaching, the morphology can be further characterized, distinguished, and confirmed. Both types of materials (especially LCs) and concentrations of RM in LC show drastic differences. These may be ascribed to the solubility limit of the monomer (or oligomer) in the LC host, which has a control over the diffusion rate or the reaction rate. Overall, we hope that the results presented in this paper could help improve understanding of the mechanism for surface localized stabilization and further extend the feasibility to combine surface localized stabilization and photo-alignment for liquid crystal optical device fabrication.

## Figures and Tables

**Table 1:** When studying the RM concentration, mixtures of RM (Bis-MA) /LC (ZLI-4792) were prepared as below. In each mixture, the added amount of PI was 1% with respect to the RM.

Sample	RM/LC (Weight Ratio)	UV Intensity (mW/cm^2^)	UV Curing Time (min)	UV Curing Temperature (°C)
RMC1	1.5:98.5	3.5	10	22
RMC2	1:99	3.5	10	22
RMC3	0.5:99.5	3.5	10	22

**Table 2:** For study on the types of liquid crystals, 1.5% RM257 was added to 2 different LC (either E7 or ZLI-4792). In each mixture, the added amount of PI was 1% with respect to the RM.

Sample	LC	UV Intensity (mW/cm^2^)	UV Curing Time (min)	UV Curing Temperature (°C)
LC1	E7	3.5	10	22
LC2	ZLI-4792	3.5	10	22

**Table 3:** With regard to the types of the reactive monomer, two different reactive monomers—either RM257 or Bis-MA were added to ZLI-4792 at 0.5%. In each mixture, the added amount of PI was 1% with respect to the RM.

Sample	RM	UV Intensity (mW/cm^2^)	UV Curing Time (min)	UV Curing Temperature (°C)
RM1	RM257	3.5	10	22
RM2	Bis-MA	3.5	10	22

**Table 4:** With regard to the PI concentration, different concentrations of PI (with respect to the RM) were prepared. In each cell, the weight ratio of RM to LC was kept constant (RM257/ZLI-4792 = 0.3/99.7).

Sample	PI/RM (Weight Ratio)	UV Intensity (mW/cm^2^)	UV Curing Time (min)	UV Curing Temperature (°C)
PI1	1:100	3.5	10	22
PI2	50:100	3.5	10	22
PI3	75:100	3.5	10	22
PI4	100:100	3.5	10	22

**Table 5:** A previous study presented one UV intensity (3.5 mW/cm^2^ at 365 nm for 10 min) for RM polymerization [34]. To see whether UV intensity will affect the surface localized polymerization, we cured the cells at different UV intensities and different times, which is shown in Table 5. In each mixture, the added amount of PI was 1% with respect to the RM.

Sample	RM/LC (Weight Ratio)	UV Intensity (mW/cm^2^)	UV Curing Time (min)	UV Curing Temperature (°C)
UVI1	0.3:99.7	0.75	70	22
UVI2	0.3:99.7	3.5	10	22
UVI3	0.3:99.7	21	2.5	22
UVI4	0.3:99.7	21	10	22

**Table 6:** With regard to the UV curing temperature, cells filled with 1.5% RM257 dissolved in E7 were UV cured at two temperatures (below and above the clearing point of E7), which is shown below. In each mixture, the added amount of PI was 1% with respect to the RM.

Sample	RM/LC (Weight Ratio)	UV Intensity (mW/cm^2^)	UV Curing Time (min)	UV Curing Temperature (°C)
UVT1	1.5:98.5	3.5	10	22
UVT2	1.5:98.5	3.5	10	80

**Table 7:** Calculation process of the solubility parameter based on the molecular structure of FLC#1.

Components for FLC #1	Cohesive Energy = Ej (J/mol)	Molar Volume = Vj (cm^3^/mol)	# of Group = Nj	Cohesive Energy Density Ec,j = Ej/Vj (J/cm^3^)	Volume = Vj × Nj (cm^3^/mol)	Volume Fraction = Vf = Vj × Nj/(Volume Sum)	Component Solubility Parameter = Ec,j^0.5^ × Vf (J/cm^3^)^0.5^
-CH_2_-	4940	16.1	4	306.8	64.4	0.27	4.7
6 atom ring	1050	16	2	65.6	32	0.13	1.1
Phenylene	31,940	52.4	1	609.5	52.4	0.22	5.4
C-F_3_	4270	57.5	1	74.3	57.5	0.24	2.1
CH_3_-	4710	33.5	1	140.6	33.5	0.14	4.2
					Volume sum = 239.8		Solubility parameter sum δ = 17.5

**Table 8:** Calculation process of the solubility parameter based on the molecular structure of FLC#2.

Components for FLC #2	Cohesive Energy = Ej (J/mol)	Molar Volume = Vj (cm^3^/mol)	# of Group = Nj	Cohesive Energy Density = Ec,j = Ej/Vj (J/cm^3^)	Volume = Vj × Nj (cm^3^/mol)	Volume Fraction = Vf = Vj × Nj/(Volume Sum)	Component Solubility Parameter = Ec,j^0.5^ × Vf (J/cm^3^)^0.5^
-CH_2_-	4940	16.1	2	306.8	32.2	0.11	2.0
-O-	3350	3.8	1	881.6	3.8	0.01	0.4
phenylene (tetrasubstituted)	31,940	14.4	2	2218.1	28.8	0.10	4.8
phenylene	31,940	52.4	1	609.5	52.4	0.18	4.6
-CF_2_-	4270	23	1	185.6	23	0.08	1.1
F- (trisubstituted)	2300	22	3	104.5	66	0.24	2.4
F- (disubstituted)	3560	20	2	178	40	0.14	1.9
CH_3_-	4710	33.5	1	140.6	33.5	0.12	1.4
					Volume sum = 279.7		Solubility parameter sum δ = 18.6

**Table 9:** Solubility parameters of RM and LC materials used in the experiments.

Materials	RM257	Bis-MA	E7	ZLI-4792
Solubility parameters (J/cm^3^)^0.5^	22.81 [37]	18.35 [37]	22.19 [38]	17.5–18.6

## References

[1] Yaroshchuk O., Reznikov Y. (2012). Photoalignment of liquid crystals: Basics and current trends. J. Mater. Chem..

[2] Ichimura K. (2000). Photoalignment of Liquid-Crystal Systems. Chem. Rev..

[3] O’Neill M., Kelly S. (2000). Photoinduced surface alignment for liquid crystal displays. J. Phys. D. Appl. Phys..

[4] Yaroshchuk O., Gurumurthy H., Chigrinov V.G., Kwok H.S., Hasebe H., Takatsu H. (2007). Photoalignment Properties of Brilliant Yellow Dye. Proc. IDW.

[5] Gao K., Cheng H., Bhowmik A.K., Bos P.J. (2015). Thin-film Pancharatnam lens with low f-number and high quality. Opt. Express.

[6] Finnemeyer V., Bryant D., Reich R., Clark H., Berry S., Bozler C., Yaroshchuk O., Lu L., Bos P. (2015). Versatile alignment layer method for new types of liquid crystal photonic devices. J. Appl. Phys..

[7] Su L., Wang B., West J., Reznikov Y. (2001). Liquid Crystal Photoalignment on Azo-Dye Layers. Mol. Cryst. Liq. Cryst. Sci. Technol. Sect. A Mol. Cryst. Liq. Cryst..

[8] Chigrinov V., Muravski A., Kwok H.S., Takada H., Akiyama H., Takatsu H. (2003). Anchoring properties of photoaligned azo-dye materials. Phys. Rev. E.

[9] Yaroshchuk O., Kyrychenko V., Tao D., Chigrinov V., Kwok H.S., Hasebe H., Takatsu H. (2009). Stabilization of liquid crystal photoaligning layers by reactive mesogens. Appl. Phys. Lett..

[10] Mcginty C., Finnemeyer V., Reich R., Clark H., Berry S., Bos P. (2017). Stable azodye photo-alignment layer for liquid crystal devices achieved by ‘turning off’ dye photosensitivity. J. Appl. Phys..

[11] Mcginty C., Wang J., Finnemeyer V., Reich R., Clark H., Berry S., Bos P. (2018). Highly Versatile and Stable Photoalignment Process for AMLCDs. SID Symp. Dig. Tech. Pap..

[12] Dierking I., Kosbar L.L., Lowe A.C., Held G.A., Dierking I., Kosbar L.L., Lowe A.C., Held G.A. (1997). Network morphology of polymer stabilized liquid crystals. Appl. Phys. Lett..

[13] Jakli A., Kim D.R., Chien L.C., Saupe A., Chien C. (1992). Effect of a polymer network on the alignment and the rotational viscosity of a nematlc llquld crystal. J. Appl. Phys..

[14] Yu M., Wang L., Nemati H., Yang H., Bunning T., Yang D.K. (2017). Effects of polymer network on electrically induced reflection band broadening of cholesteric liquid crystals. J. Polym. Sci. Part B Polym. Phys..

[15] Yamaguchi R., Inoue K., Kurosawa R. (2016). Effect of Liquid Crystal Material on Polymer Network Structure in Polymer Stabilized Liquid Crystal Cell. J. Photopolym. Sci. Technol..

[16] Zhang H., Cao H., Chen M., Zhang L., Jiang T., Chen H., Li F., Zhu S., Yang H. (2017). Effects of the fluorinated liquid crystal molecules on the electro-optical properties of polymer dispersed liquid crystal films. Liq. Cryst..

[17] Yin Y., Li W., Cao H., Guo J., Li B., He S., Ouyang C., Cao M., Huang H., Yang H. (2009). Effects of Monomer Structure on the Morphology of Polymer Network and the Electro-optical Property of Reverse-Mode Polymer-Stabilized Cholesteric Texture. J. Appl. Polym. Sci..

[18] Sonin A.S., Churochkina N.A. (2010). Liquid crystals stabilized by polymer networks. Polym. Sci. Ser. A.

[19] Rajaram C.V., Hudson S.D., Chien L.C. (1995). Morphology of Polymer-Stabilized Liquid Crystals. Chem. Mater..

[20] Wang Q., Ma Z., Li H., Li X., Huang X. (2000). Effects of curing temperature and UV intensity on electro-optical properties of PSCT reverse-mode light shutters. Proc. SPIE.

[21] Rajaram C.V., Hudson S.D., Chien L.C. (1996). Effect of Polymerization Temperature on the Morphology and Electrooptic Properties of Polymer-Stabilized Liquid Crystals. Chem. Mater..

[22] Rajaram C.V., Hudson S.D., Chien L.C. (1998). Morphology of diacrylate copolymer networks formed in liquid crystalline media. Polymer.

[23] Sonin A.S., Churochkina N.A. (2013). Liquid crystals stabilized by physical networks. Polym. Sci. Ser. A.

[24] Dierking I. (2010). Recent developments in polymer stabilised liquid crystals. Polym. Chem..

[25] Dierking I. (2000). Polymer network-stabilized liquid crystals. Adv. Mater..

[26] Dierking I. (2014). A review of polymer-stabilized ferroelectric liquid crystals. Materials.

[27] Kang S.W., Sprunt S., Chien L.C. (2002). Photoinduced localization of orientationally ordered polymer networks at the surface of a liquid crystal host. Macromolecules.

[28] Vorflusev V., Kumar S. (1999). Phase-Separated Composite Films for Liquid Crystal Displays. Science.

[29] Qian T., Kim J.H., Kumar S., Taylor P.L. (2000). Phase-separated composite films: Experiment and theory. Phys. Rev. E.

[30] Lu L., Sergan V., Bos P.J. (2012). Mechanism of electric-field-induced segregation of additives in a liquid-crystal host. Phys. Rev. E.

[31] Wang J., McGinty C., West J., Bryant D., Finnemeyer V., Reich R., Berry S., Clark H., Yaroshchuk O., Bos P. (2017). Effects of humidity and surface on photoalignment of brilliant yellow. Liq. Cryst..

[32] Chigrinov V., Kwok H.S., Takada H., Takatsu H., Chigrinov V., Kwok H.O.I.S., Takada H., Takatsu H. (2005). Photo-aligning by azo-dyes: Physics and applications. Liq. Cryst. Today.

[33] Chigrinov V., Pikin S., VePrudnikova E., Kozenkov V., Khazimullin M., Ho J., Huang D.D., Kwok H.S. (2004). Diffusion model of photoaligning in azo-dye layers. Phys. Rev. E.

[34] Mcginty C., Finnemeyer V., Reich R., Clark H., Berry S., Bos P. (2017). Strong Effect of Azodye Layer Thickness on RM-Stabilized Photoalignment. SID Symp. Dig. Tech. Pap..

[35] Ma R., Yang D. (2000). Freedericksz transition in polymer-stabilized nematic liquid crystals. Phys. Rev. E..

[36] Brandrup J., Immergut E.H., Grulke E.A. (1999). Polymer Handbook.

[37] Lu L. (2012). controllable Liquid Crystal Alignment with the Assistance of Reactive Monomers. Ph.D. Thesis.

[38] Jung J.A., Kim B.K. (2005). Controls of solubility parameter and crosslinking density in polyurethane acrylate based holographic polymer dispersed liquid crystal. Opt. Commun..

